# Beyond the Bedside: The Pivotal Role of Nursing Clinical Leadership in Developing Virtual Ward Care Through Telemedicine—A Review of the Evidence

**DOI:** 10.1111/jocn.70413

**Published:** 2026-06-20

**Authors:** Clare Lewis, Thomas Kearns, Michael Shannon

**Affiliations:** ^1^ Faculty of Nursing and Midwifery Royal College of Surgeons Ireland Dublin Ireland; ^2^ Global Leadership Innovation Academy Faculty of Nursing and Midwifery Royal College of Surgeons Ireland Dublin Ireland

**Keywords:** integration, leadership, nursing, telehealth, telemedicine, virtual wards

## Abstract

**Aim:**

To synthesise international evidence on virtual wards (VWs) models and examine the pivotal role of clinical nursing leadership in the development, governance, and delivery of digitally enabled acute care.

**Background:**

Virtual wards deliver hospital‐level care in patients' homes through telemedicine including remote monitoring, supporting admission avoidance and early supported discharge. While existing literature largely focuses on clinical outcomes and medical oversight, the contribution of nursing leadership to VW development and sustainability remains underexplored.

**Design and Methods:**

An integrative review was conducted, synthesising evidence from empirical studies, service evaluations, and policy literature. Searches were undertaken between February and April 2025 across CINAHL, PubMed, EMBASE, and the Cochrane Library, alongside targeted grey literature searches. The review focused on acute, hospital‐integrated VW models implemented in Australia, Ireland, and the United Kingdom, enabling comparison of nursing leadership roles across three publicly funded healthcare systems.

**Results:**

Twenty‐four sources met inclusion criteria, with six studies selected for in‐depth case analysis. Across all models, nurses, particularly those in senior and advanced practice roles, were central to VW implementation. Nursing leadership underpinned key enablers of successful VW delivery, including staff training, clinical governance frameworks, standardised protocols, interoperable electronic health records, and patient empowerment through technology. Nurses led remote assessment and monitoring, coordinated multidisciplinary teams, operationalised escalation pathways, and ensured continuity of care across acute and community settings.

**Conclusion:**

This review demonstrates that clinical nursing leadership is foundational to the effectiveness, safety, and scalability of VWs. While medical leadership provides essential diagnostic oversight, nursing leadership delivers sustained operational coordination, patient‐centred design, and system integration. Empowering nurses to lead VW development is therefore critical to advancing integrated, high‐quality acute care beyond the hospital setting.

**Statement:**

No patient or public contribution.

## Abstract (highlights)

**What Problem Did This Review Address?:**

This review examined the development of telemedicine enabled VW models and addressed the limited understanding of how clinical nursing leadership contributes to the design, governance, and delivery. Specifically, it explores how nurses can lead the integration of telemedicine and new models of acute, community‐hospital integrated care to improve patient outcomes beyond traditional inpatient settings.

**What Were the Main Findings?:**

Virtual Wards were shown to enhance healthcare efficiency, access, and continuity of care, particularly for acute medical conditions managed in the home and for reducing pressure on inpatient and outpatient services. Across models from Australia, Ireland, and the United Kingdom, key enablers of successful VW implementation included nurse‐led staff training, robust clinical governance frameworks, standardised protocols, interoperable Electronic Health Records (EHRs), and patient empowerment supported by remote technologies. These enablers were consistently operationalised through nursing leadership roles.

**Where and on Whom Will the Research Have an Impact?:**

The findings have direct relevance for clinical nursing leadership across acute, community, and integrated care settings. The review provides actionable insights for nurse leaders at all levels to develop and implement VW models, including shaping integrated care pathways, leading telemedicine adoption, coordinating multidisciplinary teams, ensuring workforce development, and advocating for appropriate governance structures, digital infrastructure, and resources.

**What Does This Paper Contribute to the Wider Global Clinical Community?:**

This review strengthens the international evidence base supporting VWs as a safe and effective alternative to acute hospital care, particularly in extending hospital‐level services into patients' homes. By highlighting the role of clinical nursing leadership, the paper demonstrates how nurses act as key drivers of digital healthcare integration, service coordination, and patient‐centred design within VW models. It highlights that the sustainability and scalability of virtual care depend not only on technology, but on skilled nursing leadership capable of delivering, governing, and evolving these services across health systems.

**What This Paper Contributes to the Wider Global Clinical Community:**

- This integrative review synthesises evidence from Australia, Ireland, and the United Kingdom, highlighting how clinical nursing leadership underpins the development and delivery of digitally enabled integrated VW models in the person's home.
- The findings demonstrate that nursing leadership is central to the operationalisation of VWs, including remote assessment and monitoring, clinical governance, escalation pathways, and interdisciplinary coordination across hospital and community settings.
- For nursing practice, this review identifies significant opportunities for leadership and innovation, requiring expanded competencies in digital literacy, remote clinical decision‐making, and coordination of complex care, positioning nurses as key leaders in the future of virtual acute care.

## 1 Introduction

The global pandemic significantly accelerated the adoption of VW models, establishing them as a cornerstone of contemporary healthcare reform and providing alternative pathways to acute hospital care, including inpatient and emergency services (Smithson et al. 2021; Greenhalgh et al. 2021; Hutchings et al. 2021; O'Malley et al. 2022; Nunan et al. 2020; Ferry et al. 2021). A VW is broadly defined as a model of care that delivers hospital‐level clinical assessment, monitoring, and treatment to patients in their own homes or community settings, supported by remote monitoring technologies, and structured clinical governance arrangements (Dhalla et al. 2014; Leung et al. 2015; Lewis et al. 2017; Low et al. 2017; Kanyimo 2022; Shi et al. 2024; Westby et al. 2024). Virtual wards (VWs) are designed to replicate key functions of an inpatient ward, including regular clinical review, escalation pathways, and coordinated multidisciplinary care, while enabling admission avoidance or early supported discharge (Dhalla et al. 2014; Lewis et al. 2017; Greenhalgh et al. 2021; NHS England 2024). Central to this model is the integration of advanced remote monitoring technologies, enabling clinicians to assess, monitor, and respond to patient needs at a distance (Health Innovation Network 2021; Cushen et al. 2022; Doherty et al. 2022). Telemedicine includes a range of technologies across health and social care (Greenhalgh et al. 2017, 2021) forming the bedrock of virtual care, frequently leveraging wearable devices to track physiological parameters and detect early deterioration (Flodgren et al. 2015). Evidence from a Cochrane review demonstrates that remote technologies that support clinical interventions can achieve outcomes comparable to in‐person care across a range of settings (Flodgren et al. 2015).

VWs have evolved into strategic solutions for managing patient volumes, optimising resources, alleviating inpatient strain, and reducing healthcare‐associated infection risks, while also demonstrating improved patient‐reported quality of life and a clear patient preference for VW care over acute hospital admission (Department of Health 2021; Health Innovation Network 2023). This paradigm shift to community focused care, extending hospital‐level care into the patient's home through a VW has already yielded substantial results; for instance, in the UK, NHS England reports that over 332,742 people have been cared for at home rather than in a hospital since May 2022, providing an extra 9.2% capacity for bed space, with plans to reach 16%–20% of the bed base (NHS England 2024). This has economic implications in the development of VWs, particularly those using digitally enabled care through remote monitoring, showing significant reduction in healthcare costs by lowering hospital admissions, shortening lengths of stay, and decreasing emergency department visits, offering a cost‐effective alternative to traditional inpatient care across a range of clinical conditions (Department of Health 2021; Health Innovation Network 2021, 2023; NHS England 2024). This includes conditions such as acute heart failure, with evidence that telehealth (a range of technologies including remote monitoring technology) aided outpatient management of acute heart failure in a specialist VW compared with standard care (without remote monitoring) reduced in re‐hospitalisations at 1, 3, 6, and 12 months compared to standard care (Sankaranarayanan et al. 2024). This also includes lower mortality rates compared to standard care (without remote monitoring), reported as 5% mortality rates (554 patients) compared to 13.7% for standard care (Sankaranarayanan et al. 2024).

While traditionally focused on acute and long‐term conditions with proven success (Dhalla et al. 2014; Low et al. 2017; Lewis et al. 2017; Sankaranarayanan et al. 2024), VWs are expanding into diagnostic outpatient care demonstrating broad efficiency benefits including a reduction in outpatient waiting times from 6‐weeks to 1‐week for hypertension monitoring (Health Innovation Network 2021; Jalilian et al. 2024). In improving access to services and patient choice, the evidence shows that patients access to VWs and remote technology yielded high satisfaction rates with patients opting for VW care rather than acute hospital care (Leung et al. 2015; Department of Health 2021; Health Innovation Network 2021; Norman et al. 2023; Jalilian et al. 2024). Moreover, patients receiving VW care experience fewer hospital admissions and are able to remain at home without needing acute hospital services (Leung et al. 2015; Health Innovation Network 2021; Norman et al. 2023; Jalilian et al. 2024; Sankaranarayanan et al. 2024), while also using less healthcare overall when compared to control groups receiving standard care from general practitioners and primary care teams for similar conditions and symptoms (Health Innovation Network 2021). There is also evidence of improvements in function, quality of life, and self‐management (Leung et al. 2015; Department of Health 2021; Health Innovation Network 2021, 2023). Crucially, VWs foster innovation, enabling new models of care through advanced telemedicine approaches (Health Innovation Network 2021, 2023). Ultimately, this integrated service delivery approach ensures that VWs provide high‐quality, hospital‐level care in the home, supported by appropriate technology and a well‐trained, accessible clinical workforce, all while promoting patient‐centred care and shared decision‐making (NICE 2023).

Recent evidence syntheses examined inpatient‐level care at home delivered through VWs with a focus on patient safety, hospital use, outcomes and cost efficiency (Shi et al. 2024). Results indicated that mortality rates or hospital admissions did not increase in patients accessing VWs. Notably, Shi et al. (2024) observed that some lower‐technology VW and hospital‐at‐home models relying on hospital and community‐based healthcare professionals supported by basic telemedicine rather than continuous monitoring demonstrated comparable safety outcomes and potential cost advantages. The review highlighted the importance of workforce configuration and clinical leadership over technology intensity alone (Shi et al. 2024). Additionally, a rapid review by Westby et al. (2024) highlighted that the effectiveness of a VW is shaped by service configuration, maximising on workforce roles and skill mix, patient selection, and alignment with local health system capacity.

While these reviews provide valuable insights into VW design, effectiveness, and implementation mechanisms, neither explicitly examines the role of clinical nursing leadership in the development, governance, and operational delivery of telemedicine‐enabled VW services (Shi et al. 2024; Westby et al. 2024). In particular, the contribution of nurses and advanced nursing roles in coordinating care, leading remote monitoring, shaping governance frameworks, and integrating telemedicine into routine practice remains underexplored. This integrative review addresses this gap by synthesising international evidence from Australia, Ireland, and the United Kingdom, with a specific focus on the leadership role of nurses in advancing VW care.

## 2 Methods

### 2.1 Design

An integrative review methodology was adopted to enable the synthesis of diverse forms of evidence, including empirical research, service evaluations, and policy and grey literature. Integrative reviews are particularly suited to examining complex healthcare interventions and service delivery models where context, implementation processes, and professional roles are central to understanding how care is developed and delivered (Whittemore and Knafl 2005). This methodology was selected to explore the development of VW models and the role of nursing leadership, rather than to evaluate the effectiveness of a single intervention. Given that the evidence base for VWs and digitally enabled care is still developing and varied, the integrative review methodology is especially well‐suited for exploration of this subject matter (Whittemore and Knafl 2005).

The reporting of this integrative review was guided by the principles of the Preferred Reporting Items for Systematic Reviews and Meta‐Analyses (PRISMA) 2020 statement (Page et al. 2021). This was used as a reporting guide rather than as a prescriptive methodological framework. Elements specific to systematic reviews, such as formal risk‐of‐bias assessment or meta‐analysis, were not applied, as the aim of this review was to synthesise diverse evidence to explore service models, implementation processes, and the role of clinical nursing leadership in VW care. This approach aligns with established guidance for integrative reviews, where structured reporting frameworks are used to enhance transparency while allowing methodological flexibility appropriate to complex service delivery research (Whittemore and Knafl 2005).

A PRISMA flow diagram (Figure 1) illustrates the identification, screening, eligibility, and inclusion of records. Searches were conducted iteratively across multiple academic databases and targeted grey literature sources.

**FIGURE 1:**
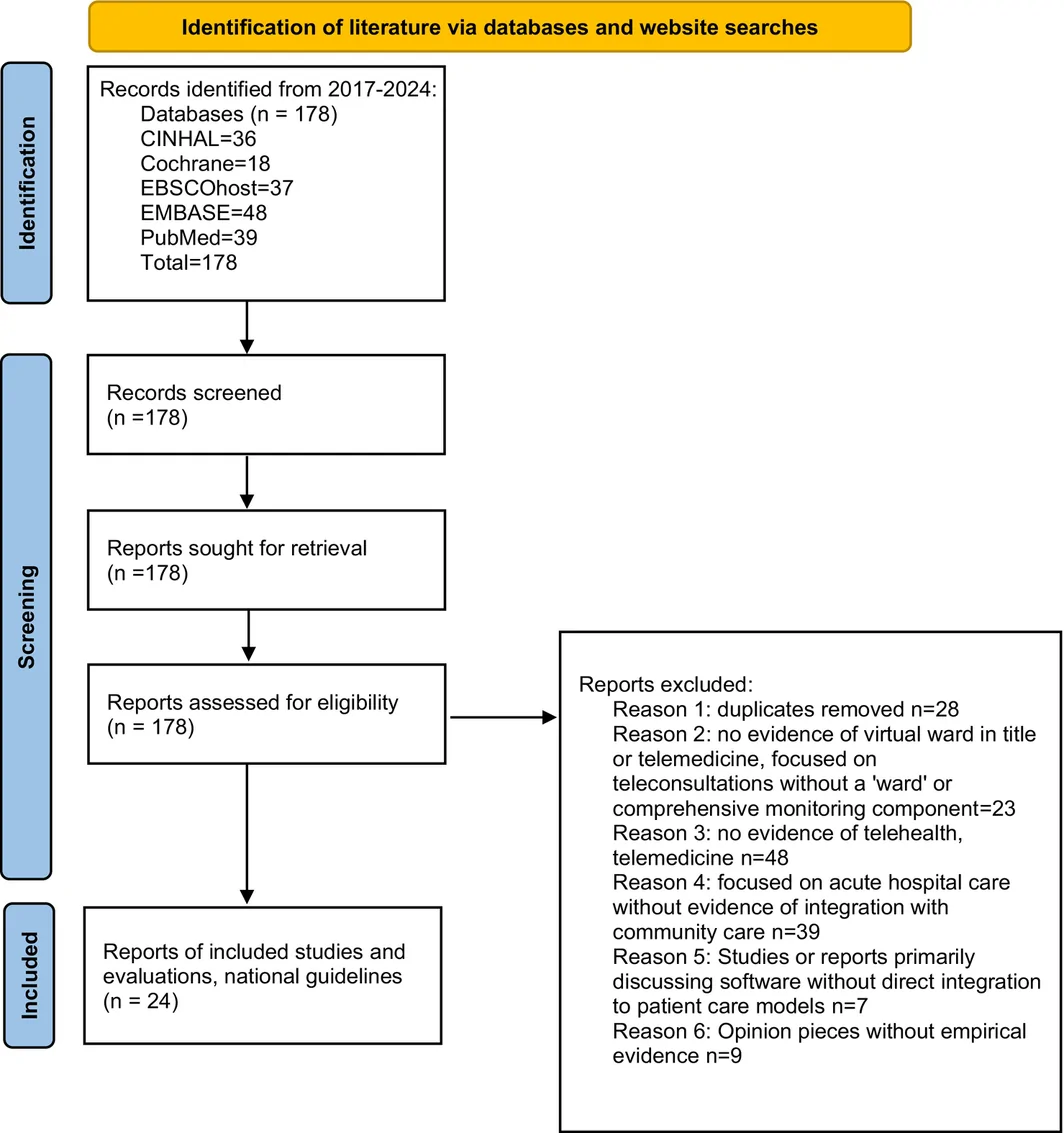
PRISMA flow diagram. Duplicate records were identified at multiple stages due to iterative database and grey literature searching, consistent with integrative review methodology. [Colour figure can be viewed at wileyonlinelibrary.com]

As is common in integrative reviews that incorporate policy documents and service evaluations, duplicate records were identified at more than one stage of the screening process. In particular, overlap occurred between database searches and targeted website searches of national health and innovation bodies, resulting in some duplicate records being identified during full‐text review rather than exclusively at the initial screening stage.

### 2.2 Search Strategy and Data Sources

A systematic and reproducible search strategy was developed to identify literature relating to VW models and clinical nursing leadership. Searches were conducted between February and April 2025 across the following databases: CINAHL (via the EBSCOhost platform), PubMed, EMBASE, and the Cochrane Library.

Search terms were adapted for each database and combined using Boolean operators (AND, OR), truncation, and parentheses. Controlled vocabulary was used where available, including Medical Subject Headings (MeSH) in PubMed and equivalent subject headings in CINAHL and EMBASE, to enhance search sensitivity.

The search strategy was structured around key concepts relating to VW models, hospital‐at‐home care, nursing, and clinical leadership. Technology‐related terms (e.g., telemedicine, telehealth, remote monitoring, and digital health) were incorporated to enhance search sensitivity; however, they were not used to limit retrieval to studies explicitly indexed under telemedicine or telehealth, thereby minimising the risk of excluding relevant VW literature.

An example of the PubMed search strategy is provided below:

(“virtual ward*” OR “hospital at home” OR “home hospital*” OR “community virtual ward*”)

AND

(nurs* OR “advanced practice nurs*” OR “clinical leader*” OR leadership)

AND

(telemedicine OR telehealth OR “remote monitoring” OR “digital health” OR “remote consultation”).

Reference lists of included studies were hand‐searched to identify additional relevant literature. Targeted grey literature searches were also undertaken across government, health policy, and innovation websites in Australia, Ireland, and the United Kingdom

The final sample reflects records that met the predefined inclusion criteria and contributed to the synthesis of evidence on VW models and the role of clinical nursing leadership in telemedicine‐enabled care delivery.

### 2.3 Inclusion and Exclusion Criteria

The inclusion and exclusion criteria were applied to ensure that the review focused on VW models delivering acute or hospital‐level care beyond the traditional inpatient setting, supported by remote monitoring technologies, and integrated with community or outpatient services. While VW terminology is used inconsistently across the literature, sources were included only where a structured ‘ward‐like’ model was evident, incorporating ongoing clinical oversight, escalation pathways, and multidisciplinary coordination.

In line with the aim of this review, studies and reports were further selected based on their relevance to service development, implementation, governance, and workforce roles, with particular attention to evidence that explicitly or implicitly presented the contribution of clinical nursing leadership. Sources that focused solely on standalone teleconsultations, technological platforms without care delivery context, or non‐acute community services were excluded. The inclusion criteria prioritised studies offering explicit or implicit insights into clinical and nursing leadership within community‐hospital integrated VW models (Table 1).

**TABLE 1:** Inclusion and exclusion criteria applied to identify technology‐enabled, community‐hospital‐integrated VW models relevant to clinical nursing leadership.

Inclusion criteria	Exclusion criteria
Focused on VW models and included technology enabled care programmes	Focused on acute hospital‐based care only Opinion pieces without empirical evidence
Discussed service delivery and implementation	Studies, evaluations or reports solely focused on teleconsultations without a ‘ward’ or comprehensive monitoring component
Discussed key challenges and enablers	Studies, evaluations or reports primarily discussing hardware/software without direct application to patient care models.
Demonstrated outcome and impact	
Provided insights relevant to clinical leadership, particularly nursing leadership, within the context of virtual care	

### 2.4 Data Extraction and Synthesis

Data included in the literature were extracted and charted. Key information extracted included country of origin, specific VW model description, patient populations, technological components, service delivery structure, identified barriers and challenges, key enablers, reported outcomes (clinical, patient experience, economic), and explicit or implicit references to the role of nursing and clinical leadership. Through iterative comparative analysis of the six in‐depth case studies, recurring leadership‐related patterns were identified and synthesised into five overarching themes. These themes are presented in Table 4, and represent the key domains through which clinical nursing leadership shapes VW development and delivery.

## 3 Results

A total of 24 sources met the inclusion criteria and informed the overall synthesis (Table 2). Of these, six studies were selected for in‐depth case analysis based on the richness of reported data relating to VW implementation, technology‐enabled integration, leadership roles, and service outcomes. This approach enabled detailed examination of nursing leadership within complex VW models while retaining a broader contextual understanding of the evidence base. The selection of these six studies was guided by the depth and relevance of information they provided across key domains: implementation strategies (detailing the practical steps and considerations involved in delivering VWs), challenges and barriers (identifying common obstacles encountered during implementation and operation), key enablers (highlighting factors that facilitated effective technology‐enabled care delivery), and outcomes and impact, with a particular emphasis on the role of nursing in this evolving model of care.

**TABLE 2:** Virtual ward sources (*n* = 24).

Author(s)/year	Country	Study type/design
Ferry et al. (2021)	Australia	Retrospective cohort study (COVID‐19 VW)
Hutchings et al. (2021)	Australia	Observational cohort study (COVID‐19 VW)
Health Innovation Network (2021)	UK	Rapid evaluation (Croydon VW)
Health Innovation Network (2023)	UK	Pan‐London evaluation report
Cushen et al. (2022)	Ireland	Service evaluation (Respiratory CVW)
Doherty et al. (2022)	Ireland	Proof‐of‐concept study (COPD CVW)
Department of Health (2021)	Ireland	Service evaluation/policy report
Dhalla et al. (2014)	Canada	Randomised clinical trial
Greenhalgh et al. (2021)	UK	Service evaluation (COVID‐19 VW support)
Jalilian et al. (2024)	UK	Retrospective longitudinal study
Kanyimo (2022)	UK	Quality improvement report
Ko et al. (2023)	Singapore	Qualitative study
Leung et al. (2015)	Hong Kong	Pilot study
Lewis et al. (2017)	Ireland	Community VW evaluation study
Low et al. (2017)	Singapore	Randomised controlled trial
NHS England (2024)	UK	Operational framework
NICE (2023)	UK	Health technology evaluation
Norman et al. (2023)	UK	Rapid evidence synthesis
Nunan et al. (2020)	UK	Service evaluation (COVID pathway)
O'Malley et al. (2022)	UK	Retrospective clinical evaluation
Sankaranarayanan et al. (2024)	UK	Comparative telehealth study
Shi et al. (2024)	International	Systematic review and meta‐analysis
Sunny et al. (2025)	International	Scoping review
Westby et al. (2024)	UK	Rapid review

*Note:* The table lists all 24 sources included in the integrative review. Six studies were selected for in‐depth case analysis based on the richness of implementation and nursing leadership data.

Focusing on these six studies and evaluations from Australia, the UK and Ireland (Table 3) enabled the extraction of rich, actionable insights that can directly inform the design, delivery, and scaling of telemedicine services within VW models. While the included studies described a range of VW models and implementation approaches, the synthesis focused specifically on identifying how nursing leadership shaped the development, governance, and delivery of care. Across the case studies, nurses, particularly those in advanced and senior roles, were central to coordinating multidisciplinary teams, leading clinical decision‐making, and integrating digital technologies into patient‐centred care pathways.

**TABLE 3:** Synthesises international VW case studies, foregrounding the leadership functions undertaken by nurses in the development, governance, and delivery of telemedicine‐enabled care models.

Country	Model/study	Key features (incl. outcomes)	Implementation challenges	Key enablers	Nursing leadership role in the VW model
Australia	Ferry et al. (2021) COVID‐19 VW	Hospital‐based VW; remote care risk‐stratified triage; nurse‐led monitoring; medical officer discharge review; median LOS 8 days; 92% managed at home; hospital admission 1.9%	Reliance on basic home devices; self‐reported symptoms; diagnostic uncertainty	Defined escalation protocols; MDT working; standardised assessment pathways	Nurses led initial triage, conducted structured remote assessments, monitored symptom progression, activated escalation pathways, and coordinated MDT communication, enabling safe hospital avoidance
Australia	Hutchings et al. (2021) Community VW	Community‐based VW for COVID‐19; early warning systems; real‐time remote monitoring; median 16 contacts per patient; low escalation and admission rates	Technology usability; fragmented communication systems; administrative burden	Remote monitoring devices; staff training; video consultations	Nurses led daily clinical monitoring, interpreted remote data, conducted holistic assessments (clinical and psychosocial), educated patients on technology use, and coordinated escalation to medical staff
UK	Croydon Virtual Ward	7‐day service; home monitoring kits and wearables; real‐time dashboards; 65% admission avoidance; significant cost savings (£742 per patient); early detection of deterioration	Manual data collection; equipment logistics; staff training in continuous monitoring	Integrated digital platforms; shared care records; clear escalation pathways	Senior nurses provided daily clinical oversight, interpreted continuous monitoring data, coordinated MDT responses, managed remote monitoring logistics, and ensured governance across acute–community boundaries
UK	Pan‐London VW Evaluation	Multiple acute and community VWs; spot and continuous monitoring; nursing as primary patient contact; 70%–99% remained at home; high patient satisfaction	Variable clinician buy‐in; inconsistent VW definitions; technical setup issues	Standardised protocols; staff upskilling; EHR access	Nursing teams acted as the primary coordinators of care, leading remote monitoring, patient engagement, escalation decision‐making, and cross‐sector communication
Ireland	Community VW (Cushen et al. 2022)	Nurse‐led respiratory VW; remote monitoring platform; ANP–consultant collaboration; daily VWrounds; cost €88/day vs. €820 hospital	Fragmented data systems; paper‐based documentation; coding challenges	Co‐designed staff training; shared governance frameworks; role clarity	ANPs led clinical decision‐making, coordinated hospital–community integration, supervised community nurses, and ensured governance and continuity of care
Ireland	CVW for COPD (Doherty et al. 2022)	ANP‐led model; continuous respiratory monitoring; digital platform integration; avoided admissions; mean saving €14,999 per patient	Implementation requirements included the need for proactive escalation pathways; data driven decision support	Seamless data integration; specialist–generalist MDT model	ANPs provided daily data‐driven reviews, led escalation decisions, coordinated multidisciplinary input, and empowered patients through education and self‐management

### 3.1 Australia Virtual Ward Implementation

Two Australian studies provide empirical evidence that VWs, supported by telemedicine, can safely and effectively deliver hospital‐level care beyond traditional inpatient settings (Ferry et al. 2021; Hutchings et al. 2021). Importantly, these studies illustrate how nursing leadership was central to the design, operationalisation, and governance of these models, rather than merely contributing to the delivery. Across both settings, nurse‐led triage, assessment, monitoring, and escalation processes enabled admission avoidance, reduced unnecessary hospitalisation, and supported system‐wide capacity management (Ferry et al. 2021; Hutchings et al. 2021).

Both studies reported low ambulance conveyance rates (3%; 5/162 patients), high rates of hospital avoidance (92%; 205/223 patients), and low hospital admission rates (1.9%–8%), outcomes that were closely linked to nursing‐led monitoring, risk stratification, and escalation decision‐making (Ferry et al. 2021; Hutchings et al. 2021).

### 3.2 Australian Study 1: Pandemic Remote Monitoring

This Australian study (Ferry et al. 2021) reported on a VW model treating 223 patients diagnosed with COVID‐19 (March–May 2020), with a median age of 45 years. Patients were admitted from community and hospital pathways and received remote care in the community delivered following standardised protocols (Ferry et al. 2021).

#### 3.2.1 Model and Care Delivery

The VW service relied on patient self‐reporting and remote monitoring technologies to inform stratification coupled with the patient's age, comorbidities, and symptom burden, directly informing consultation frequency (Ferry et al. 2021). While medical officers retained responsibility for diagnostic review and discharge decisions, nurses led the operational application of this risk stratification model, conducting structured remote assessments, monitoring symptom trajectories, and activating escalation pathways when deterioration was identified.

Nurses were typically the first point of contact, undertook initial assessments, and coordinated escalation to medical review where predefined criteria were met. This nurse‐led surveillance and decision‐making enabled timely intervention, including hospital referral when required, while maintaining patient safety within a home‐based care model (Ferry et al. 2021).

An in‐person assessment was informed by an escalation criteria including

1. The patient reported severe symptoms related to shortness of breath, cough, or fatigue.
2. Symptoms became more severe either on 1 observation of patients aged > 65 years with comorbidities or over 2 observations in those without comorbidities.
  Or
3. Staff or patient concerns regarding exacerbation. If required, hospital referral was arranged for further assessment.

All patients underwent a final teleconsultation review by a medical officer; however, nurses led the coordination of multidisciplinary care and discharge planning, acting as the primary point of contact to ensure continuity and readiness for discharge (Ferry et al. 2021).

The median length of stay was 8 days (range 1–44 days), and several factors were correlated to a length of stay > 7 days. These included having any comorbidity classified as high risk on admission or being hospitalised prior to VW admission (Ferry et al. 2021).

#### 3.2.2 Implementation Challenges

A key challenge was relying on basic remote monitoring devices (e.g., household thermometers), making clinical data largely dependent on patient self‐reporting during telephone consultations (Ferry et al. 2021). This limitation reduced diagnostic certainty in the remote setting and may have lowered the threshold for hospital referral (Ferry et al. 2021). Importantly, this challenge highlights a leadership opportunity for nurses to advocate for investment in more sophisticated remote monitoring technologies that strengthen nurse‐led assessment and escalation within VW models (Ferry et al. 2021).

#### 3.2.3 Key Enablers

Key enablers supporting the VW included established agreed escalation pathways to acute hospital services. Additionally, the development of clear protocols, roles, and responsibilities was crucial for effective care delivery, supported by a systematic approach to patient assessment (Ferry et al. 2021). Nursing leadership was instrumental in the development, implementation, and consistent application of these protocols, ensuring clarity of responsibility, continuity of care, and safe escalation processes within the VW model (Ferry et al. 2021).

#### 3.2.4 Outcomes and Impact

Of the 223 patients managed within the VW, 205 (92%) were successfully cared for at home and discharged following clinical recovery, without requiring a hospital admission. Only 18 (8%) patients required a hospital referral, with one third (six patients) subsequently discharged after assessment in the emergency department, indicating effective nurse‐led remote triage and escalation decision making. Worsening, ongoing, or new‐onset dyspnea was the most common trigger for escalation. The high rate of successful home management highlights the model's safety and effectiveness, demonstrating its potential to enhance inpatient capacity, reduce healthcare‐associated infections, and validate nursing‐led monitoring and early intervention strategies (Ferry et al. 2021).

### 3.3 Australian Study 2: Community‐Based Virtual Ward Programme

This 12‐month Australian pilot VW programme, initially designed for chronic conditions, was strategically repurposed for patients diagnosed with COVID‐19, enrolling 162 individuals (93.6% of total, median age 38) from the community (Hutchings et al. 2021).

#### 3.3.1 Model and Care Delivery

Operating as a community‐based service, the VW was underpinned by a formal clinical governance framework, with nurses leading day‐to‐day clinical monitoring, assessment, and coordination of care (Hutchings et al. 2021). Nurses applied a structured assessment approach encompassing clinical status, psychosocial wellbeing, and functional capacity, informed by real‐time remote monitoring data.

Through frequent virtual contacts, predominantly via video consultation, nurses interpreted physiological data, identified early signs of deterioration, and coordinated escalation to medical officers when required. This nurse‐led monitoring and communication infrastructure functioned as the operational backbone of the VW model (Hutchings et al. 2021) (see Figure 2 for specific assessment items).

**FIGURE 2:**
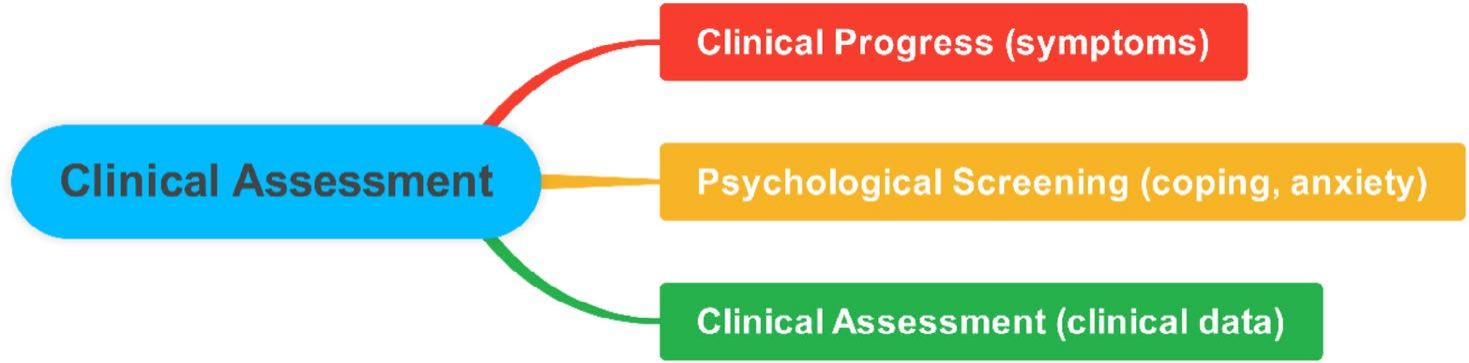
Nurse‐led assessment. [Colour figure can be viewed at wileyonlinelibrary.com]

This model was augmented by nurse‐led use of medical devices that provided real‐time data on oxygen saturation, temperature, heart rate, and blood glucose for patients with diabetes, alongside structured assessment of mental state and fluid intake (Hutchings et al. 2021). Nurses interpreted data during frequent video consultations, which constituted the primary mode of clinical assessment during admission, and coordinated escalation to medical officers for teleconsultation review and remote prescribing when deterioration was identified. Through this nurse‐led monitoring and communication framework, patients were safely managed and discharged after approximately seven days from illness onset, following clinical improvement, a negative PCR test, and 48 h without fever (Hutchings et al. 2021).

#### 3.3.2 Implementation Challenges

Barriers to delivery included patient and caregiver difficulties with technology use, fragmented communication systems, and technical issues affecting consultation workflows (Hutchings et al. 2021). Addressing these challenges required active nursing leadership in problem‐solving, staff education, patient support, and advocacy for streamlined digital systems capable of supporting safe and efficient virtual care delivery.

#### 3.3.3 Key Enablers

Key enablers included enhanced assessment capabilities through remote monitoring devices and comprehensive staff training (Hutchings et al. 2021). Nursing leadership played a central role in designing and delivering this training, supporting workforce competence and empowering patients and caregivers to engage confidently with remote monitoring technologies.

#### 3.3.4 Outcomes and Impact

This technology enabled VW demonstrated safe hospital avoidance, with low escalation and admission rates. These outcomes were underpinned by nurse‐led monitoring, assessment, and escalation decision‐making, reinforcing the central role of nursing leadership in achieving safe, effective hospital‐at‐home care (Hutchings et al. 2021).

### 3.4 UK Virtual Wards Leveraging Technology

In the United Kingdom, VWs are increasingly leveraging technology to deliver hospital‐level care in patients' homes, with extensive development, implementation, and evaluation across the health system (Health Innovation Network 2021, 2023; NICE 2023; NHS England 2024). This represents a transformative shift in care delivery, in which clinical nursing leadership has played a pivotal role in shaping service design, operational models, and governance arrangements (Health Innovation Network 2021, 2023; NHS England 2024). Two comprehensive UK papers, a Pan‐London evaluation of VWs and an evaluation of the Croydon VW, are presented to demonstrate this (Health Innovation Network 2021, 2023).

### 3.5 Croydon Virtual Ward

The evaluation of the Croydon Virtual Ward illustrates how nursing leadership underpins technology‐enabled VW models (Health Innovation Network 2021). The model expanded an established rapid response community service, with remote monitoring significantly enhancing its capacity to safely manage patients at home.

#### 3.5.1 Model and Care Delivery

The model operated within a 7‐day 8 am–8 pm service supported by a multidisciplinary team (Health Innovation Network 2021) and provided active monitoring of up to 30 patients. Referrals were accepted from acute hospitals, primary care, and community services for a range of acute medical conditions, including cardiac and respiratory illness (Health Innovation Network 2021). Patients were predominantly older adults (≥ 80 years) with multiple comorbidities, requiring high levels of clinical oversight. Nursing leadership was integral to managing this complexity and ensuring continuity of care within a home‐based, technology‐enabled model (Health Innovation Network 2021).

While medical staff provided diagnostic oversight, nurses led day‐to‐day operational management of the VW, overseeing remote monitoring, interpreting physiological data, coordinating care, and activating escalation pathways. Data were transmitted to a web‐based dashboard designed to replicate inpatient observation charts, with predefined thresholds generating alerts for clinical review (Health Innovation Network 2021). Patients were provided with home monitoring kits comprising wearable devices capturing data such as oxygen saturation, respiratory rate, heart rate, skin temperature, and movement during the day and overnight.

Nurses referred to this data alongside symptom assessment during telephone and video consultations, which formed the basis of daily patient management. Changes in clinical status or patient‐reported concerns prompted nurse‐led escalation, including home visits by the rapid response team when required. Through this nurse‐led monitoring and coordination model, timely intervention was achieved while maintaining patient safety and continuity of care. The average length of stay was 11.5 days (median 6 days) (Health Innovation Network 2021).

#### 3.5.2 Implementation Challenges

Implementation challenges included manual data entry, administrative burden, and logistical complexities associated with the delivery and retrieval of monitoring equipment (Health Innovation Network 2021). Additional challenges related to workforce upskilling, particularly in interpreting continuous versus spot monitoring data and adapting to new modes of clinical decision‐making. These challenges highlight the critical leadership role of nurses in advocating for automated digital solutions, standardised data integration, and sustained investment in nursing informatics and workforce development (NHS England 2024).

#### 3.5.3 Key Enablers

Key enablers included infrastructure supporting real‐time data sharing between the VW, acute hospital services, and community teams through shared care records (Health Innovation Network 2021). Comprehensive training and ongoing support for staff and patients were essential to ensure safe and confident use of remote monitoring technologies. Integrated escalation pathways with clear routes to acute services further supported patient safety. Nursing leadership was central to the development and operationalisation of these enablers, including pathway design, workforce training, equipment logistics, and advocacy for interoperable digital systems that underpin effective virtual care delivery (Health Innovation Network 2021).

#### 3.5.4 Outcomes and Impact

Despite implementation challenges, the Croydon VW demonstrated that technology‐enabled, nurse‐led coordination of care can deliver safe, effective, and cost‐efficient alternatives to inpatient admission (Health Innovation Network 2021; Jalilian et al. 2024). A total of 65% of patients (162/250) avoided hospital admission, with estimated savings of £742 per patient and total savings of £185,610 across the cohort. The reported cost savings were based on reduced contact and bed‐day costs rather than a full service‐level costing that explicitly itemises staffing and operational expenses. Continuous remote monitoring enabled early detection of serious pathologies, including pulmonary embolism and cardiac arrhythmias, supporting timely intervention and potentially preventing adverse outcomes (Health Innovation Network 2021).

Patients managed within the VW demonstrated lower healthcare utilisation, reduced outpatient appointments, and shorter diagnostic waiting times, including reduction of hypertension diagnostic pathways from 6 weeks to 1 week (Health Innovation Network 2021). Improvements in patient confidence, independence, and quality of life were also reported, with patients describing care as equivalent or superior to hospital‐based treatment (Health Innovation Network 2021). Collectively, these outcomes highlight the value of nursing leadership in enabling VWs to function as integrated, patient‐centred systems of care rather than isolated technological interventions.

### 3.6 UK Pan‐London Virtual Ward Evaluation

A comprehensive UK Pan‐London evaluation of VWs across six South London boroughs offers crucial insights into how telemedicine integrated into VWs is revolutionising patient care (Health Innovation Network 2023). This evaluation examined the impact of VWs on reducing hospital admissions and facilitating early discharge, demonstrating a shift of hospital level care into the community (Health Innovation Network 2023). Importantly, it also explored key enablers, patient engagement with technology, user experiences, and outcomes that were closely linked to deployment of technologies and shaped by local clinical leadership, with nursing leadership playing a central operational role across sites (Health Innovation Network 2023).

#### 3.6.1 Model and Care Delivery

Virtual Wards in South London were built upon digital health approaches, primarily utilising remote monitoring and remote monitoring devices (Health Innovation Network 2023). Conditions managed included acute infections, acute kidney injury, atrial fibrillation, acute respiratory illness, and decompensating heart failure. (Health Innovation Network 2023). Clinical governance varied across boroughs, encompassing acute led, primary care led, and acute care led community delivered models. Despite this variation, nursing teams consistently led day‐to‐day operational delivery, acting as the primary point of contact for patients, coordinating multidisciplinary input, and overseeing remote monitoring and escalation processes (Health Innovation Network 2023).

Nurses monitored physiological parameters including temperature, respiratory rate, heart rate, blood pressure, and oxygen saturation, using telemedicine platforms to support clinical decision‐making. Through this nurse‐led coordination of monitoring and virtual engagement, continuous oversight and timely therapeutic intervention were maintained. Length of stay ranged from 1 to 29 days, with average stays of 7, 10, and 13 days across boroughs (Health Innovation Network 2023).

#### 3.6.2 Implementation Challenges

Several barriers were reported, including a lack of awareness and buy‐in, and low clinician awareness of VW models, which affected patient onboarding (Health Innovation Network 2023). This was potentially linked to inconsistencies in the definition of VWs, affecting standardised implementation (Health Innovation Network 2023). Technical challenges and issues with continuous monitoring access were also noted. Addressing these systemic and technical barriers required strong nursing leadership to support clear communication, advocate for standardised protocols, and lead change management initiatives across diverse clinical environments (Health Innovation Network 2023).

#### 3.6.3 Key Enablers

Patients reported positive experiences with technology, supported by caregiver involvement, technical assistance, and high levels of virtual communication (Health Innovation Network 2023). Workforce upskilling, particularly among nursing staff, was identified as a critical enabler for effective use of continuous and spot monitoring technologies. Clear protocols for remote and in‐person care supported safe and consistent service delivery, while access to electronic health records (EHRs) enabled seamless care coordination and informed clinical decision‐making (Health Innovation Network 2023). These enablers highlight the leadership role of nurses in developing competency frameworks, leading training initiatives, and advocating for integrated digital systems to support sustainable virtual care (NHS England 2024).

#### 3.6.4 Outcomes and Impact

The evaluation showed reduced hospital re‐admissions, reporting that 70%–99% remained at home (including data from over 5000 patients) (Health Innovation Network 2023). Improvements in patient function and self‐management were also reported, empowered by remote support (Health Innovation Network 2023). The lower need for in‐person visits did not compromise high patient satisfaction (Health Innovation Network 2023). These outcomes reflect the effectiveness of VWs and nurse‐led coordination, adaptive clinical practices, and interdisciplinary collaboration within Pan‐London VW models (Health Innovation Network 2023).

### 3.7 Ireland Advancing Home‐Based Acute Care Through Virtual Wards

From an Irish perspective, the literature highlights the growing role of VWs supported by technology, in transforming the delivery of acute care in the home. These initiatives consistently demonstrate the central role of nursing leadership in pioneering, operationalising, and sustaining nurse‐led VW models within integrated hospital–community systems (Department of Health 2021; Cushen et al. 2022; Doherty et al. 2022).

### 3.8 Community Virtual Ward for Respiratory Care

Irish Community Virtual Wards (CVWs) represent a significant step in leveraging technology to transform acute care delivery in a community setting (Department of Health 2021; Cushen et al. 2022). These initiatives illustrate how nurse‐led virtual care pathways can safely support patients with acute and chronic respiratory conditions while avoiding unnecessary hospital admission. (Department of Health 2021; Cushen et al. 2022).

#### 3.8.1 Model and Care Delivery

A proof‐of‐concept nurse‐led CVW model (20 virtual beds) illustrated the impact of digital health when embedded within a structured nursing leadership framework (Department of Health 2021; Cushen et al. 2022). The model was underpinned by a remote monitoring platform and remote monitoring devices measuring peak flow, oxygen saturation, and pulse rate for continuous/spot‐monitoring of respiratory patients (Department of Health 2021; Cushen et al. 2022). This enabled patients predominantly aged ≥ 65 years with multiple co‐morbidities to receive close monitoring while avoiding hospital admission (Department of Health 2021; Cushen et al. 2022).

Referrals were received from general practitioners, respiratory outpatient and inpatient services. The CVW operated a 7‐day 8 am–8 pm nurse‐led CVW with nursing leadership co‐ordinating care delivery. A substantial portion of patient care was delivered remotely, averaging 5.5 virtual contacts over an 11‐day admission, supplemented by 2–3 home visits. Community nurses conducted an initial home visit to assess the environment and inform remote monitoring plans, and these were integrated into care plans led by Advanced Nurse Practitioners (ANPs) based in the acute hospital (Department of Health 2021; Cushen et al. 2022).

Daily CVW ward rounds facilitated structured communication between community nurses and hospital‐based ANPs to ensure continuity and shared clinical decision‐making. Shared governance arrangements were established between the acute hospital and community organisation, and a respiratory consultant provided clinical governance, clinical support and attended CVW ward rounds remotely (Department of Health 2021). An electronic platform enabled information sharing, access to screening tools, and visibility of patient data across settings. Escalation pathways to the acute hospital were informed by data generated from remote technologies, enabling timely speciality input whilst avoiding an unplanned hospital admission. (Department of Health 2021; Cushen et al. 2022). Nursing staff received dedicated training in remote monitoring and virtual care delivery to support a shift to virtual healthcare delivery (Department of Health 2021; Cushen et al. 2022).

#### 3.8.2 Implementation Challenges

Challenges reported included fragmented data systems, reliance on paper‐based systems increasing administrative burden (Department of Health 2021). Coding challenges and unstructured data were also noted affecting the ability to measure outcomes and impact, highlighting the need for standardising data input and generating structured data from telemedicine systems (Department of Health 2021). These challenges highlight the importance of nursing leadership in driving digital transformation initiatives that are standardised and support clinical practice efficiently and effectively (Department of Health 2021).

#### 3.8.3 Key Enablers

Key enablers included staff training and education in remote care management, interpretation of remote monitoring data, use of telemedicine platforms, and virtual consultation skills (Department of Health 2021; Cushen et al. 2022). Training was co‐designed with nurses and technical specialists, emphasising the nursing leadership role in virtual care development. Role clarity to support interdisciplinary collaboration, with standardised protocols and defined roles supported by structured communication pathways supporting collaboration between ANPs, community nursing, acute hospital teams, and primary care (Department of Health 2021; Cushen et al. 2022). These arrangements were underpinned by shared governance frameworks with nursing leadership central to establishing a safe, co‐ordinated, and integrated model of care (Department of Health 2021; Cushen et al. 2022).

#### 3.8.4 Outcomes and Impact

The CVW pilot demonstrated improved patient outcomes, enhanced self‐management and quality of life using remote monitoring and virtual support (Department of Health 2021; Cushen et al. 2022). Patients remained at home with an average length of stay of 11 days (Department of Health 2021; Cushen et al. 2022) supported during acute exacerbations, with structured escalation pathways to outpatient day services if required. Reduced anxiety and increased health literacy were also reported with patients attributed to improved understanding of their condition to the virtual care model (Department of Health 2021; Cushen et al. 2022). When compared to acute hospital care, the CVW admission was estimated at €88/day versus €820/day for acute hospital (€969 vs. €9020 total). Projections indicated annual savings of over €15.9 million if operating three CVWs across regions, highlighting the potential of VWs to optimise healthcare expenditure (Department of Health 2021). These outcomes highlight the effectiveness of nurse‐led leadership in delivering safe, high‐quality, and economically sustainable VW care (Department of Health 2021).

### 3.9 Ireland Community Virtual Ward and Continuous Remote Monitoring for COPD

A further Irish study provides strong evidence of a nurse‐led Community Virtual Ward (CVW) model and the impact of technology in managing COPD exacerbations in the community (Doherty et al. 2022). This model demonstrates how advanced nursing leadership can safely deliver specialist respiratory care at home through the integration of continuous remote monitoring technologies. The CVW utilised continuous respiratory rate monitoring (cRR) alongside a digital health platform (myPatientSpace) integrated with wearable oximetry (RespiraSense), spirometry, and non‐invasive pulse oximeters to support comprehensive, real‐time clinical monitoring (Doherty et al. 2022).

#### 3.9.1 Model and Care Delivery

Within this model, an ANP led the daily operational and clinical management of patients through systematic review and interpretation of remote monitoring data. Changes in physiological trends triggered nurse‐led virtual reviews, risk reassessment, and timely initiation of rescue prescriptions, enabling early intervention and preventing hospital admission (Doherty et al. 2022). Through continuous tracking of respiratory parameters and other clinical indicators, the ANP exercised autonomous clinical decision‐making, coordinating care and triggering escalation pathways when required.

Enabled by a remote monitoring platform and wearable devices for continuous data collection, the CVW adopted a specialist and generalist care approach. Nursing leadership ensured holistic therapeutic management that addressed both acute exacerbation and longer‐term disease management, supporting patient empowerment and self‐management within the home environment (Doherty et al. 2022).

#### 3.9.2 Key Enablers and Overcoming Challenges

The effectiveness of this model was underpinned by the seamless integration of technology for continuous data capture and interpretation, alongside structured multidisciplinary collaboration (Doherty et al. 2022). A crucial enabler was the leadership role of the ANP in overcoming potential challenges, daily clinical decision‐making, managing complex patient presentations remotely, and ensuring safe coordination of care across settings (Doherty et al. 2022).

#### 3.9.3 Outcomes and Impact

The model demonstrated that COPD exacerbations could be safely managed at home, through nurse‐led, technology enabled care, avoiding hospital admission and reducing healthcare utilisation (Doherty et al. 2022). Patients reported improvements in self‐management and quality of life, reflecting the effectiveness of the CVW sustained by nurse‐led care (Doherty et al. 2022). Significant cost efficiencies were also observed with mean cost reductions of €14,999 per patient compared to usual hospital care, primarily due to avoided admissions, ambulance conveyances, and reduced hospital utilisation (Doherty et al. 2022). Collectively, these outcomes highlight the substantial impact of advanced nursing leadership in delivering safe, effective, and economically sustainable VW care.

## 4 Discussion

This integrative review demonstrates that VW models are not sustained by technology alone, but by clinical nursing leadership structures that enable telemedicine to function as a structured system of care. Across all six in‐depth case studies from Australia, Ireland, and the United Kingdom, nursing was central to the operationalisation of VWs (Ferry et al. 2021, Hutchings et al. 2021, Health Innovation Network 2021, Department of Health 2021, Cushen et al. 2022, Doherty et al. 2022). Nursing leadership consistently underpinned core functions including remote assessment and monitoring, coordination of multidisciplinary teams, management of escalation pathways, and continuity of care across acute and community interfaces. While medical leadership provides essential diagnostic oversight and supports escalation of care, the evidence demonstrates that nursing leadership delivers the continuous operational and coordination required to sustain VWs as functioning systems of care rather than episodic interventions (Ferry et al. 2021; Hutchings et al. 2021; Health Innovation Network 2021; Department of Health 2021; Cushen et al. 2022; Doherty et al. 2022).

Nursing leadership was consistently associated with key implementation functions, including risk stratification, remote assessment and monitoring, coordination of multidisciplinary teams, escalation decision‐making, and continuity of care across acute and community interfaces. In nurse‐led and nurse‐coordinated models, these leadership functions directly supported hospital avoidance, early supported discharge, and safe management of patients with acute and complex conditions in the home (Ferry et al. 2021, Hutchings et al. 2021, Health Innovation Network 2021, Department of Health 2021, Cushen et al. 2022, Doherty et al. 2022).

From the pandemic‐driven remote monitoring in Australian settings (Ferry et al. 2021; Hutchings et al. 2021) to the more established, complex care pathways in the UK's Croydon VW (Health Innovation Network 2021), nurses are central to in‐patient triage, comprehensive assessment, and continuous or spot monitoring of vital signs. They utilise digital platforms and remote monitoring devices to track patient progress, identify early signs of deterioration, and enable timely interventions or escalations to acute services (Department of Health 2021; Doherty et al. 2022; Ferry et al. 2021; Hutchings et al. 2021; Health Innovation Network 2021, 2023; Sunny et al. 2025). This essential clinical engagement by nurses ensures patient safety and smooth care transitions, allowing VWs to operate as coordinated systems of care rather than discrete episodes of remote monitoring, and to effectively manage diverse populations (Health Innovation Network 2021; Department of Health 2021).

Beyond direct clinical care, this review consistently highlights proactive nursing leadership as a critical determinant of VW success. The implementation of VW models is inherently complex, frequently encountering barriers related to technology literacy among patients and staff, complexities in data collection and interoperability, and logistical challenges associated with equipment deployment (Health Innovation Network 2021, 2023). Evidence from both UK (Health Innovation Network 2021, 2023) and Irish models (Department of Health 2021; Cushen et al. 2022; Doherty et al. 2022) demonstrates how nurse leaders have been instrumental in mitigating these challenges. Nurse leaders led targeted training initiatives, advocated for interoperable digital solutions, were involved in the co‐design of clinical governance frameworks, and oversaw the complex logistics of remote monitoring. This adaptive leadership, frequently enacted through advanced nursing roles such as ANPs, supports not only operational efficiency but also the ongoing evolution and scaling of technology‐enabled care pathways (Department of Health 2021; Doherty et al. 2022).

The evidence further highlights the impact of nurse‐led VWs on patient outcomes and healthcare system efficiency (Department of Health 2021; Doherty et al. 2022). Across the reviewed models, significant reductions in hospital admissions (Ferry et al. 2021; Hutchings et al. 2021), shorter lengths of stay, and substantial cost savings were consistently reported, as demonstrated by the Croydon VWs economic benefits (Health Innovation Network 2021) and efficiencies observed in Irish CVWs (Department of Health 2021; Doherty et al. 2022). Virtual Wards also improved patient experience by increasing access to care within the home, supporting enhanced self‐management and quality of life (Health Innovation Network 2021, 2023). The ability to detect serious pathologies earlier through continuous monitoring, as evidenced in the Croydon model, further reinforces the safety and effectiveness of this approach (Health Innovation Network 2021). These outcomes are directly attributable to the integrated clinical expertise, operational coordination, and strategic oversight provided by nursing leadership (Health Innovation Network 2021; NHS England 2024).

Collectively, the evidence demonstrates that VWs require nursing leadership to operate efficiently and effectively shaped by the clinical expertise to ensure patient safety, drive positive outcomes, and foster innovation in care delivery (Department of Health 2021; Doherty et al. 2022; Ferry et al. 2021; Hutchings et al. 2021; Health Innovation Network 2021, 2023; Sunny et al. 2025). These findings highlight the transformative potential of technology‐enabled VWs, underpinned by the central and sustained role of clinical nursing leadership in their development and success.

### 4.1 Implications for Nursing Leadership

The evidence highlights several critical domains in which clinical nursing leadership is essential to the successful implementation and sustained growth of VW models. These domains, synthesised from the case study analysis and presented in Table 4, include technology adoption, clinical governance and standardised care approaches, interdisciplinary collaboration and shared care, patient and caregiver empowerment, and advocacy for the infrastructure and resources required to sustain virtual care services.

**TABLE 4:** Implications for nursing leadership.

Virtual ward themes	Nursing leadership and opportunities
Technology adoption	
Technology adoption and competency with the successful integration hinges on both the availability of user‐friendly technology and a highly competent workforce (Health Innovation Network 2021, 2023)	Nursing has an opportunity to take a central role in proactively assessing technology needs, lead on and develop pilot programs, and ensure continuous professional development opportunities to develop digital literacy and proficiency in virtual care developing a strategic vision
Clinical governance and standardised care approaches	
Protocol development and clinical governance with clear, standardised protocols and robust clinical governance frameworks are foundational for patient safety and consistent care delivery in a virtual environment (Ferry et al. 2021; Hutchings et al. 2021, Health Innovation Network 2021, 2023, NHS England 2024)	Nurses are ideally positioned to co‐design protocols and leveraging their knowledge and skills in development of care pathways and understanding clinical risks, and to ensure their consistent application
Interdisciplinary collaboration and shared care	
Interdisciplinary collaboration and shared care with communication for effective VWs rely on seamless collaboration between nursing, medical, allied healthcare professionals, and technical support teams (Department of Health 2021; Health Innovation Network 2021, 2023)	Nursing leadership is crucial in fostering a culture of interdisciplinary teamwork, shared care, establishing effective communication channels, and bridging traditional departmental silos to optimise virtual care coordination
Patient and caregiver empowerment	
Providing education, user‐friendly technology, and ongoing support is vital for adherence and positive outcomes (Health Innovation Network 2021, 2023)	Nurses can drive initiatives focused on patient‐centred design, ensuring technology and support services meet the diverse needs of individuals receiving care at home
Infrastructure and resources	
Infrastructure and resources such as advancing electronic methods to share information and sustained funding to advance healthcare using telemedicine approaches (Department of Health 2021; Doherty et al. 2022, NHS England 2024)	Nurses are ideally positioned to act as strong advocates for the necessary digital infrastructure, funding, and staffing models required to scale and sustain VW services

In leading digital transformation and technology adoption, the development of VW models presents an opportunity for nursing leadership to extend digital competence beyond basic skills acquisition (Department of Health 2021; Health Innovation Network 2021, 2023). As illustrated in Table 4, nurse leaders are well positioned to proactively assess technology requirements, lead pilot initiatives, and embed ongoing professional development programmes (Department of Health 2021; NHS England 2024). This leadership includes developing advanced competencies in remote clinical assessment, data interpretation, telehealth communication, and the design of clinical governance frameworks specifically suited to virtual care environments (Department of Health 2021; Doherty et al. 2022; Sunny et al. 2025). Through these activities, nursing leadership transforms technological challenges into opportunities for service innovation and improved patient care, fostering organisational cultures that support adaptability and advancing clinical practice (NHS England 2024).

In relation to patient safety and clinical governance, nurses possess the clinical expertise and system knowledge required to lead and co‐ordinate the development of standardised care approaches that are foundational to safe and consistent care delivery in virtual environments (Ferry et al. 2021; Hutchings et al. 2021; Health Innovation Network 2021, 2023; NHS England 2024). As outlined in Table 4, nursing leadership plays a key role in co‐designing clinical protocols, escalation pathways, and risk management processes, and in ensuring consistent application across VW services (NHS England 2024; Sunny et al. 2025).

Interprofessional collaboration and shared care are equally central to the success of integrated VW models (Ferry et al. 2021; Hutchings et al. 2021; Health Innovation Network 2021, 2023; Department of Health 2021; Cushen et al. 2022; Doherty et al. 2022; NHS England 2024; Sunny et al. 2025). Nursing leadership is pivotal in bridging acute hospital and community services, facilitating effective collaboration among the multidisciplinary team (Department of Health 2021; Doherty et al. 2022; Health Innovation Network 2021, 2023). As highlighted in Table 4, nurse leaders foster cohesive team cultures, establish clear communication structures, and support shared clinical decision‐making processes that underpin integrated, patient‐centred care delivery (Sunny et al. 2025).

Beyond clinical and operational functions, nursing leadership extends to patient and caregiver empowerment, which is fundamental to the success of home‐based virtual care. As demonstrated in Table 4, nurses are uniquely positioned to lead patient‐centred design initiatives, ensuring technologies and support systems are accessible, usable, and responsive to individual needs (Cushen et al. 2022; Department of Health 2021; Doherty et al. 2022).

Finally, nursing leadership plays a crucial advocacy role in securing the digital infrastructure, sustained funding, and workforce models necessary to scale and sustain VW services (Department of Health 2021; Doherty et al. 2022; NHS England 2024). Nurses' integrated understanding of clinical risk, service delivery, and operational constraints positions them as key contributors to policy, research, and innovation in virtual care (Department of Health 2021; Doherty et al. 2022; Sunny et al. 2025).

## 5 Conclusion

This integrative review demonstrates that VW models represent a significant advancement in healthcare delivery, enabling the provision of hospital‐level care beyond traditional inpatient settings through the strategic use of technology, encompassing virtual consultations, remote patient monitoring, and digitally connected monitoring devices. Evidence from Australia, the United Kingdom, and Ireland highlight that the effectiveness, safety, and scalability of these models are fundamentally underpinned by clinical nursing leadership.

Drawing on the leadership domains identified in Table 4, this review illustrates how nurses play a central role in driving technology adoption, establishing clinical governance frameworks, coordinating interdisciplinary care, empowering patients and caregivers, and their role in advocating for the infrastructure and resources required to sustain VW services. While medical leadership remains essential as part of clinical governance and support escalation, nursing leadership provides the continuous operational and system‐level coordination that enables VWs to function safely and effectively at scale.

As healthcare systems continue to expand VWs, empowering nurses to lead these innovations will be crucial to ensuring quality, equity, and sustainability. The foresight, expertise, and advocacy of clinical nursing leadership will therefore remain central to the future development of VWs and the broader transformation of acute care delivery.

## Author Contributions

Dr. Clare Lewis, Professor Thomas Kearns, Professor Michael Shannon, writing, review and editing.

## Funding

The authors have nothing to report.

## Conflicts of Interest

The authors declare no conflicts of interest.

## Data Availability

Data sharing not applicable to this article as no datasets were generated or analysed during the current study.
